# Scientific Machine Learning for Polymeric Materials

**DOI:** 10.3390/polym17162222

**Published:** 2025-08-14

**Authors:** C. Fernandes

**Affiliations:** 1 CEFT—Transport Phenomena Research Center, Faculty of Engineering, University of Porto, Rua Dr. Roberto Frias, 4200-465 Porto, Portugal; cbpf@fe.up.pt; 2 ALiCE—Associate Laboratory in Chemical Engineering, Faculty of Engineering, University of Porto, Rua Dr. Roberto Frias, 4200-465 Porto, Portugal; 3 Centre of Mathematics (CMAT), University of Minho, Campus of Gualtar, 4710-057 Braga, Portugal

## 1. Introduction

Polymeric materials are ubiquitous in modern technology, from structural composites and membranes to responsive elastomers, yet their design remains challenging due to complex, multi-scale behaviors. Conventional modeling often struggles to capture phenomena spanning the molecular to the macroscopic scale in polymers. In recent years, Scientific Machine Learning (SciML), the integration of data-driven artificial intelligence (AI) with physical models, has emerged as a powerful approach to accelerate polymer research and development. By incorporating domain knowledge (such as physical laws or material science heuristics) into neural networks, SciML methods can leverage limited experimental or simulation data to build more accurate, interpretable models than black-box ML alone.

In the past five years, the emergence of SciML has transformed polymer research by combining data-driven algorithms with physical models to overcome the limitations of purely empirical or purely first-principles approaches. Graph- and sequence-based neural networks have enabled rapid screening of polymer chemistries, surrogate models and active-learning frameworks have accelerated high-throughput material discovery, and physics-informed architectures (e.g., PINNs) have begun to bridge molecular-scale dynamics with macroscopic behavior without requiring massive datasets. ML models now predict complex structure–property relationships, enabling the inverse design and high-throughput screening of polymer candidates (Xie et al. [1], Pai et al. [2]). SciML techniques now combine physics-based models with data-driven ML to enhance accuracy and interpretability, even with limited data (Audus et al. [3], Nanjo et al. [4]). ML techniques such as random forests, neural networks, and support vector machines are increasingly used to predict the thermal, mechanical, and optical properties of polymers and composites (Malashin et al. [5], Hamidi et al. [6]).

Despite these advances, critical gaps remain. Most current ML tools excel at a single length or time scale, yet polymers exhibit phenomena—from chain entanglement and phase separation to fracture and creep—that span orders of magnitude. Few SciML methods rigorously quantify prediction confidence, limiting their reliability for safety-critical or regulatory applications. Polymer data are often sparse, proprietary, or measured under inconsistent protocols, making generalization challenging. While PINNs have shown promise, they remain computationally demanding for large-scale polymer process simulations and have yet to fully incorporate complex rheological laws or multicomponent interactions. The articles in this Special Issue directly address these gaps. The multi-objective optimization of NN hyperparameters for composites and membranes (Malashin et al. [7]) demonstrates how tailored ML can capture multiple target properties simultaneously, offering a template for multi-scale model coupling. Three-dimensional autoencoder defect detection (Liu et al. [8]) and hysteresis modeling of magnetorheological elastomers (Alawi et al. [9]) illustrate how ML can embed physics constraints into high-dimension inverse problems, paving the way for more efficient, physics-driven simulators. Diffusion coefficient prediction in Nafion membranes and QSPR-based cleansing foam design showcase strategies for data-synthesis and transfer learning to mitigate data scarcity by combining experiments, surrogate models, and generative screening (Malashin et al. [10]).

This Special Issue, titled “Scientific Machine Learning for Polymeric Materials”, brings together cutting-edge work in this area, highlighting new machine learning algorithms, data-driven design strategies, and physics-informed methods tailored to polymeric systems. The collected contributions address a broad range of topics, ranging from neural network-based property prediction to advanced material characterization, illustrating the importance and promise of ML-driven approaches in polymer science.

## 2. Overview of the Published Articles

This Special Issue features several articles that apply SciML techniques to polymeric materials. Key themes include predictive modeling of polymer properties, simulation of smart polymers, and comprehensive surveys of ML methods in this field.

Recent research has showcased how ML and AI are revolutionizing polymer science—from materials design and property prediction to novel testing methods. For example, Brighel et al. [11] demonstrated that an AI-trained model can make analytical crystallization elution fractionation (aCEF) a standalone characterization tool: their model automatically classifies commercial polyolefins without any prior chemical information.

Complex polymer blends with many ingredients pose a challenge for formulation design. Hamaguchi et al. [12] tackled this by preparing 537 cleansing-foam formulations (mixtures of surfactants, polyols, etc.) and using ML to predict their cleansing performances. They computed molecular descriptors (including Hansen solubility parameters) for each formulation and trained five ML models. The best model achieved $R^{2}\approx 0.770$ in predicting cleansing capability. Importantly, the study also used in silico design: it generated and screened virtual formulations with the trained model. Despite highly nonlinear ingredient interactions, this AI-assisted approach identified promising ingredient combinations, illustrating how ML can guide the optimization of “super-multicomponent” polymer products.

Complementing these research highlights are three extensive review papers. Long et al. [13] provide an overarching perspective on AI in polymers, advocating a shift from traditional experimentation to data-driven design. They document AI’s transformative impact on polymer design, property prediction, and processing and outline strategies (e.g., collaborative data platforms, explainable models) for overcoming challenges like data scarcity. Malashin and colleagues contribute two focused reviews: one on physics-informed neural networks (PINNs) [14] in polymers and another on SVMs [15]. The PINN review summarizes recent advances in embedding physical laws into neural networks for multi-scale polymer modeling, while the SVM review surveys how kernel-based ML methods have been used to predict polymer properties, optimize synthesis, and control processes. Together, these reviews highlight the rapid integration of ML techniques, ranging from SVMs to deep PINNs, into polymer science, and they chart future directions for AI-enabled polymer innovation.

## 3. Conclusions and Outlook

In conclusion, the contributions of this Special Issue demonstrate the significant impact of advanced machine learning on polymer research. The featured studies show that integrating AI methods, ranging from neural networks and evolutionary optimization to physics-informed frameworks and ensemble learners, can greatly enhance our ability to predict, understand, and design polymeric materials. For example, the data-driven optimization of neural network models has yielded accurate predictions of composite properties, while novel 3D autoencoders have improved nondestructive defect detection in composites. Machine learning applied to polymer membranes, polymer property databases, and complex mixtures (like cleansing foams) has revealed subtle structure–property relationships and identified optimal formulations without exhaustive experimentation. The hysteresis modeling of magnetorheological elastomers illustrates how SciML can be used to capture nonlinear smart material behaviors, enabling precise device design. Complementing these technical advances, the three review articles synthesize the state of the art in the field and point toward future opportunities: they highlight, for instance, how physics-informed learning can bridge scales in polymer simulation and how boosting algorithms are reshaping data-driven polymer design.

Together, the works in this Special Issue advance polymer science by leveraging data, descriptors, and physics in novel ways. They showcase how SciML methods not only improve predictive accuracy but also offer insight into why materials behave as they do, often reducing development cycles. Looking forward, further progress will likely come from more tightly integrating physics-based knowledge with ML (as in PINNs and hybrid models), expanding open polymer datasets for training, and improving interpretability and uncertainty quantification. As noted in the Special Issue’s call for papers, the overarching goal is to use scientific AI to unravel multi-scale polymer phenomena and accelerate innovation in polymeric materials. The contributions collected here have taken important steps toward achieving that goal: they provide new models, algorithms, and frameworks that deepen our understanding and capability in polymer design. We anticipate that these advances will inform ongoing research, inspire new SciML applications in polymer science, and help to realize the Special Issue’s vision of smart, data-driven polymer innovation.

Future research should build on these foundations by developing hybrid multi-scale frameworks that seamlessly couple molecular simulations (MD/DFT) with continuum-scale ML models to predict processing–structure–property maps; embedding robust uncertainty quantification (e.g., Bayesian PINNs, ensemble learning) to deliver prediction intervals alongside point estimates, critical for design under uncertainty and regulatory approval; creating shared, standardized polymer data repositories and benchmarks that enable fair comparison, reproducibility, and meta-learning across different polymer classes and experimental conditions; optimizing physics–ML co-solvers for large-scale manufacturing simulations, leveraging reduced-order modeling, operator learning, and adaptive sampling to enable real-time digital twins of polymer processes; and expanding reinforcement-learning and generative models for closed-loop, autonomous material discovery, where AI not only predicts properties but also actively proposes and synthesizes novel polymer chemistries in laboratory workflows.
